# Supplementary material for: Xenogeneic silencing strategies in bacteria are dictated by RNA polymerase promiscuity
Source: Nat Commun. 2022 Mar 3;13:1149. doi: 10.1038/s41467-022-28747-1 (PMC8894471; doi:10.1038/s41467-022-28747-1)
Supplement: Supplementary file 1 — Supplementary Information [file 41467_2022_28747_MOESM1_ESM.pdf]

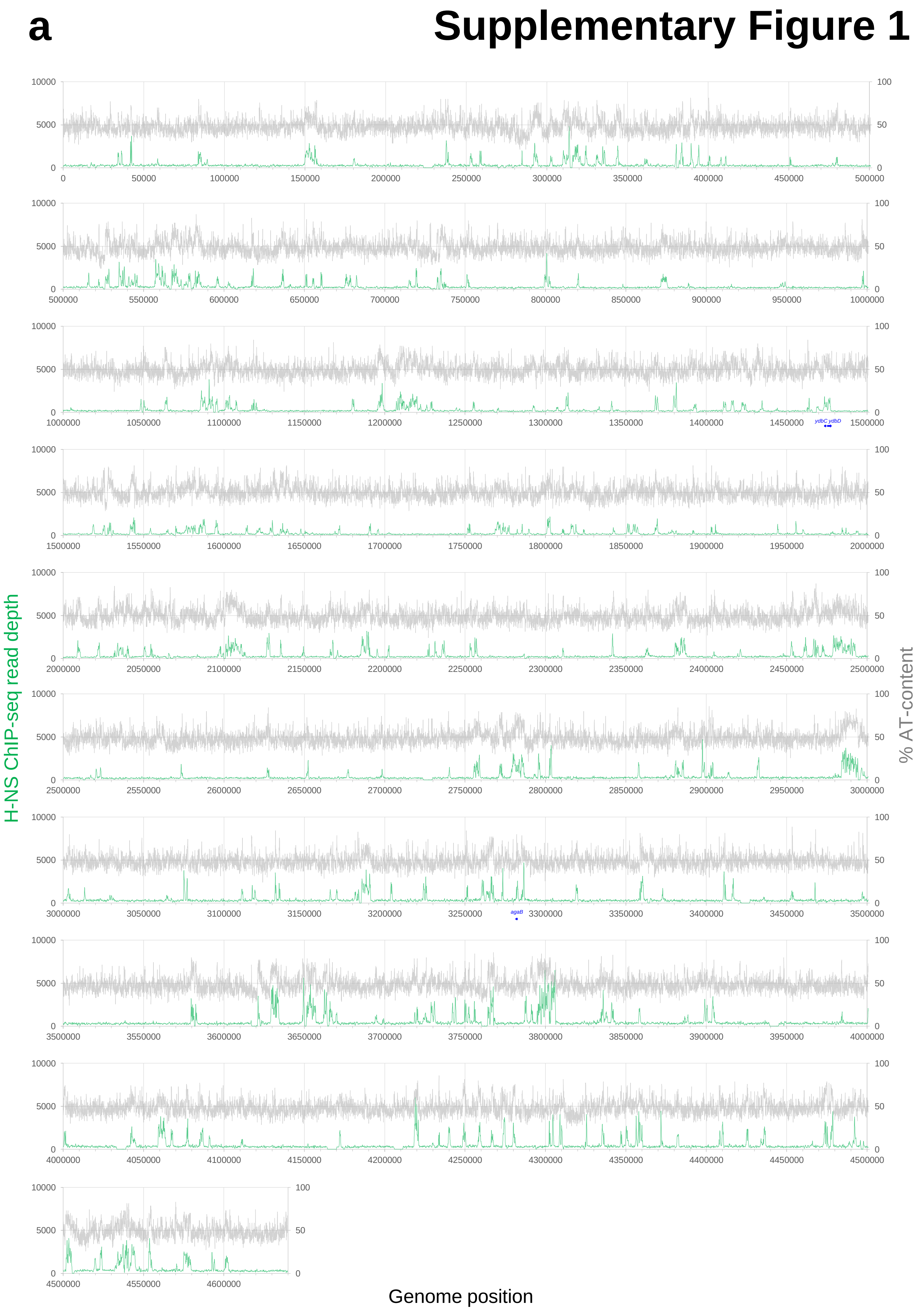
a
Supplementary Figure 1
H-NS ChIP-seq read depth
% AT-content
Genome position
ydbC ydbD
agaB

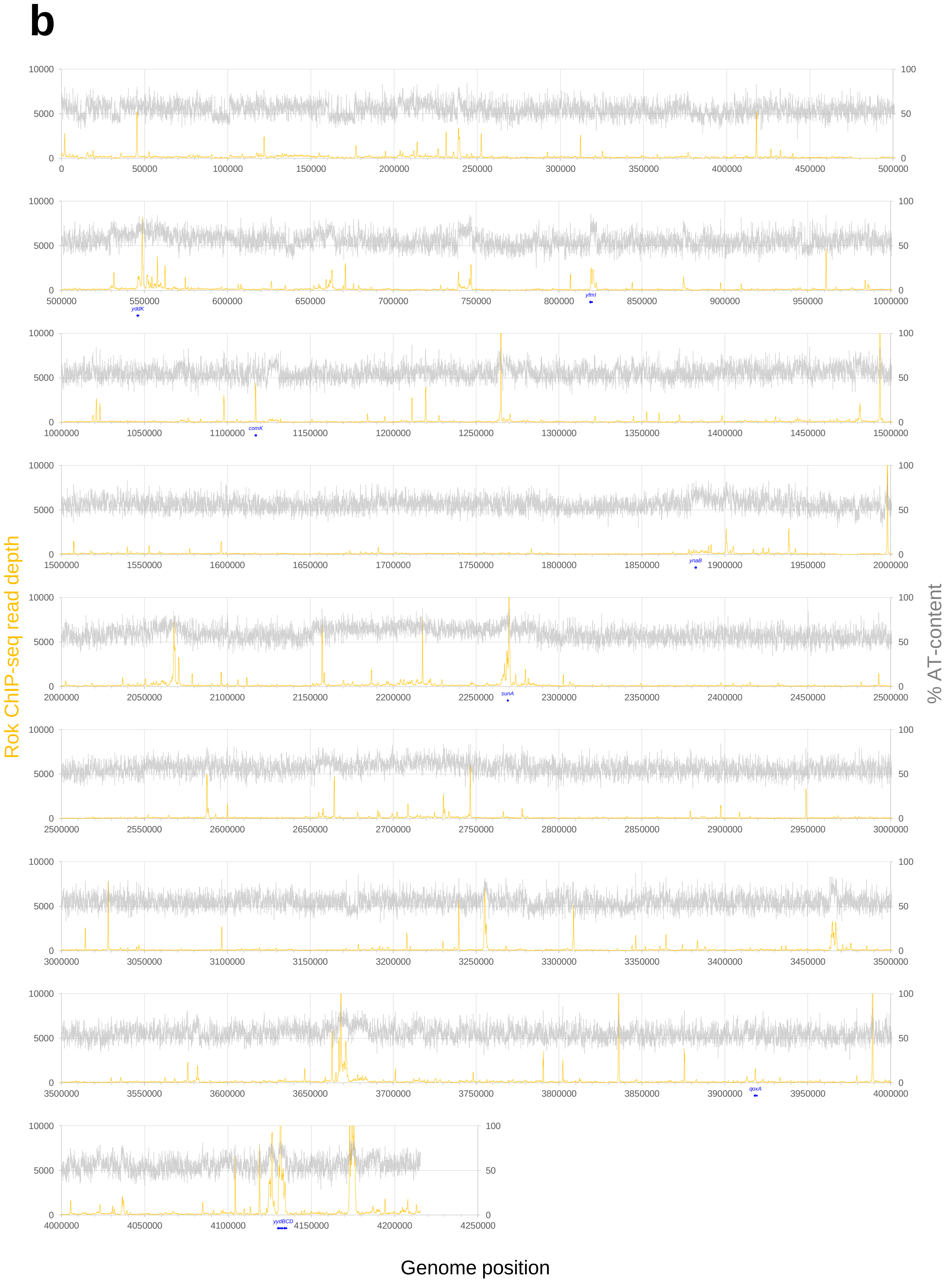
b
Rok ChIP-seq read depth
% AT-content
Genome position
10000
5000
0
100
50
0
yddK
yfmI
comK
ynaB
sunA
qoxA
yydBCD
0
500000
1000000
1500000
2000000
2500000
3000000
3500000
4000000
4250000

c

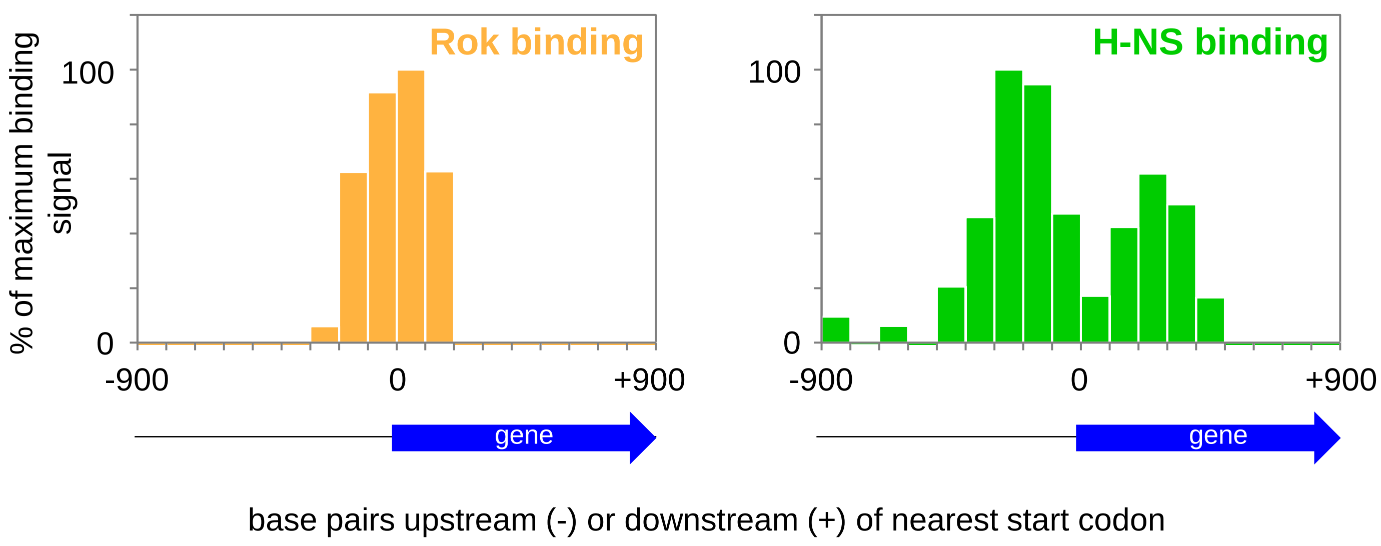
Rok binding
H-NS binding
% of maximum binding signal
100
0
-900
0
+900
gene
base pairs upstream (-) or downstream (+) of nearest start codon

d

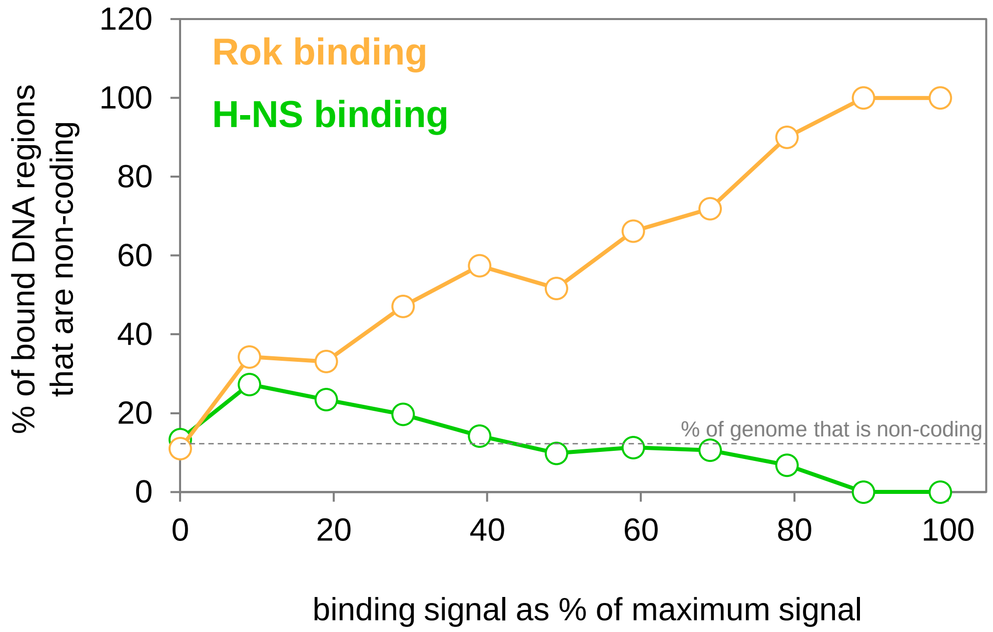
Rok binding
H-NS binding
% of bound DNA regions that are non-coding
120
100
80
60
40
20
0
% of genome that is non-coding
binding signal as % of maximum signal

Supplementary Figure 1: *E. coli* H-NS and *B. subtilis* Rok exhibit different patterns of DNA binding *in vivo*.

a) Genome-wide distribution of H-NS in *E. coli*. The H-NS ChIP-seq binding signal (green) and DNA AT-content (grey) were averaged across non-overlapping 70 bp bins and plotted against genome position. The H-NS target genes examined in this study are marked by blue arrows and labelled. Note that the H-NS ChIP-seq data were originally mapped against version U00096.2 of the *E. coli* genome[32]. Hence, we have used these coordinates in this figure panel only.

b) Genome-wide distribution of Rok in *E. coli*. The Rok ChIP-seq binding signal (orange)[35] and DNA AT-content (grey) were averaged across non-overlapping 70 bp bins and plotted against genome position. The Rok target genes examined in this study are marked by blue arrows and labelled.

c) Aggregate distribution of Rok (orange) and H-NS (green) binding signals genome-wide with respect to gene start codons. The ChIP-seq data[32,35] were averaged in 10 bp bins across the chromosome and the average signal across all bins was subtracted from each individual bin to remove background. The distance between each bin and the nearest gene start codon in the genome was then determined. Each bar shows the sum of all bins in sequential 100 bp windows upstream (-) or downstream (+) of a start codon. To aid comparison, data are presented as a % of the maximum signal for each experiment.

d) Relationship between Rok (orange) or H-NS (green) binding signal and non-coding DNA. The ChIP-seq data[32,35] were averaged in 10 bp bins across the chromosome. We then calculated the % of bins in non-coding DNA above different binding signal thresholds (shown as a percentage of the maximum binding signal). The dashed line indicates the approximate percentage of each genome that is non-coding. The absolute values are 13 % and 11 % for *E. coli* and *B. subtilis* respectively.

Supplementary Figure 2

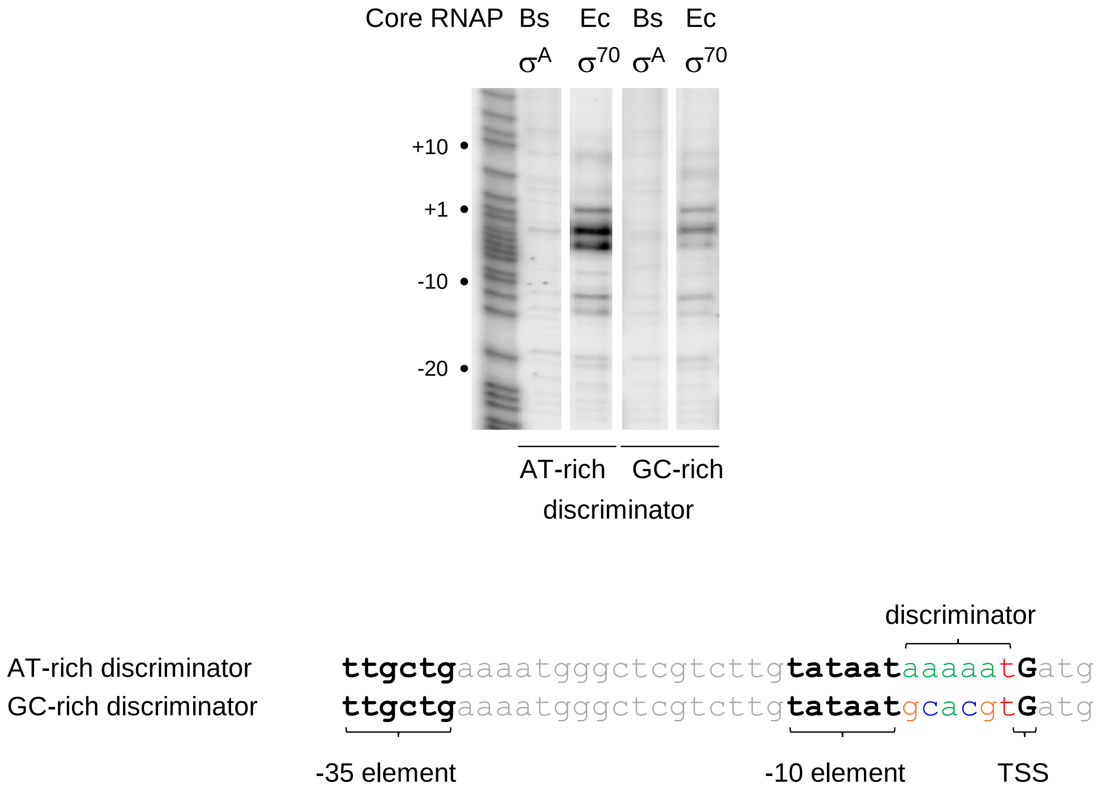

Supplementary Figure 2: Promoter discriminator sequence impacts DNA opening by *E. coli* σ^{70} and *B. subtilis* σ^{A} RNA polymerase differently. The gel image shows $KMnO_4$ reactivity patterns due to DNA opening by *E. coli* (Ec) σ^{70}, or *B. subtilis* (Bs) σ^{A}, RNA polymerase holoenzyme (0.5 µM) at the promoter sequences shown. The gel is calibrated with a Maxam-Gilbert G+A sequencing reaction. The experiment was done twice with similar results.

Supplementary Figure 3

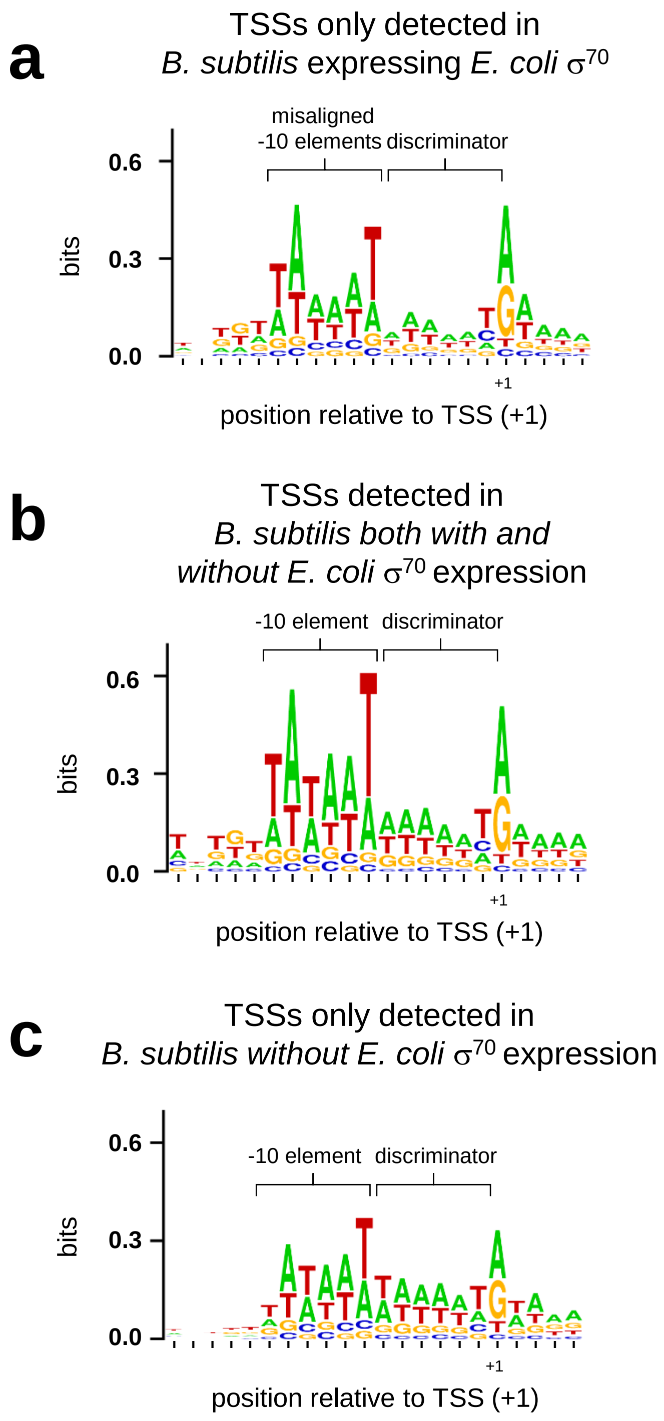

Supplementary Figure 3: *B. subtilis* transcription start sites dependent on *E. coli* σ^{70} have promoters that more closely resemble those identified in *E. coli*.

a) Positioning of promoter -10 elements and transcription start sites in *E. coli* and *B. subtilis*. The bar charts show the percentage of promoter -10 elements located at indicated distances upstream of transcription start sites identified by cappable-seq for *E. coli* and *B. subtilis*.

b) The panel shows DNA sequence logos generated by aligning nucleic acid regions upstream *B. subtilis* transcription start sites dependent on σ^{70} (left) to those also identified in the absence of σ^{70} (right). The more variable spacing between transcription start sites and promoter -10 elements at σ^{70} dependent promoters generates a motif that misrepresents the consensus -10 element sequence (5'-TATAAT-3'). The promoter discriminator sequence is less AT-rich at promoters used only by σ^{70}.

Supplementary Figure 4

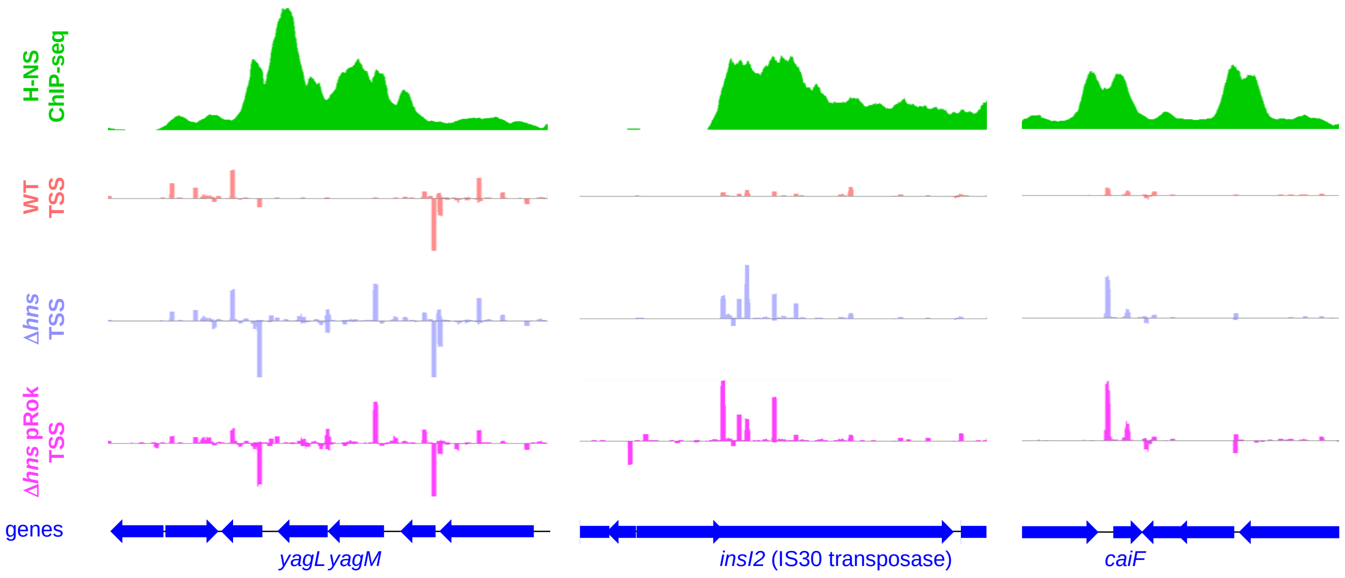

Supplementary Figure 4: Rok cannot compensate for loss of *hns* in *E. coli*. Three genomic regions subject to silencing by H-NS are shown. Data for H-NS occupancy are shown by the green graph[32]. Transcription start sites (TSSs) were identified by cappable-seq for wild type, Δ*hns*, or Δ*hns* cells carrying plasmid pRok. In the cappable-seq data only RNA 5' ends are sequenced and so the upstream edge of each peak indicates a TSS. Sequence reads mapping to the top and bottom DNA strands are shown above and below the central horizontal line in each plot. Genes are shown by blue arrows.

Supplementary Figure 5

```
sp|P06224|SIGA_BACSU      MADKQTHETELTFDQVKEQLTESGKKRGVLTYEEIAERMSSFEIESDQMDEYYEFLGEQG
sp|P00579|RPOD_ECOLI      --------MEQNPQSQLKLLVTRGKEQGYLTYAEVNDHLPEDIVDSDQIEDIIQMINDMG
                                   * . :.  : *.  **::* *** *: :::..  ::***:::  :::.: *

                                                                    100
sp|P06224|SIGA_BACSU      VELISENEETEDPNIQQLAKAEEEFDLNDLSVP----PGVKINDPVRMYLKEIGRVNLLS
sp|P00579|RPOD_ECOLI      IQVMEEAPDADDLMLAENTADEDAAEAAAQVLSSVESEIGRTTDPVRMYMREMGTVELLT
                          ::::.*  :::*  : : :  *:  :     :.       : .******::*:* *:**:

sp|P06224|SIGA_BACSU      AKEEIAYAQKIEEGD---------------------------------------------
sp|P00579|RPOD_ECOLI      REGEIDIAKRIEDGINQVQCSVAEYPEAITYLLEQYDRVEAEEARLSDLITGFVDPNAEE
                           : **  *::**:*

                                              200
sp|P06224|SIGA_BACSU      ------------------------------------------------------------
sp|P00579|RPOD_ECOLI      DLAPTATHVGSELSQEDLDDDEDEDEEDGDDDSADDDNSIDPELAREKFAELRAQYVVTR

sp|P06224|SIGA_BACSU      ------------------------------------------------------------
sp|P00579|RPOD_ECOLI      DTIKAKGRSHATAQEEILKLSEVFKQFRLVPKQFDYLVNSMRVMMDRVRTQERLIMKLCV

                                 300
sp|P06224|SIGA_BACSU      ------------------------------------------------------------
sp|P00579|RPOD_ECOLI      EQCKMPKKNFITLFTGNETSDTWFNAAIAMNKPWSEKLHDVSEEVHRALQKLQQIEEETG

                                                                    400
sp|P06224|SIGA_BACSU      --------------------EESKRRLAEANLRLVVSIAKRYVGRGMLFLDLIQEGNMGL
sp|P00579|RPOD_ECOLI      LTIEQVKDINRRMSIGEAKARRAKKEMVEANLRLVISIAKKYTNRGLQFLDLIQEGNIGL
                                              ..:*:.:.*******:****:*..**: *********:**

sp|P06224|SIGA_BACSU      MKAVEKFDYRKGYKFSTYATWWIRQAITRAIADQARTIRIPVHMVETINKLIRVQRQLLQ
sp|P00579|RPOD_ECOLI      MKAVDKFEYRRGYKFSTYATWWIRQAITRSIADQARTIRIPVHMIETINKLNRISRQMLQ
                          ****:**:**:*****************:**************:****** *:.**:**

                                              500
sp|P06224|SIGA_BACSU      DLGREPTPEEIAEDMDLTPEKVREILKIAQEPVSLETPIGEEDDSHLGDFIEDQEATSPS
sp|P00579|RPOD_ECOLI      EMGREPTPEELAERMLMPEDKIRKVLKIAKEPISMETPIGDDEDSHLGDFIEDTTLELPL
                          ::********:** *  :.  :*:*::****:**:*:*****:::**********     *

sp|P06224|SIGA_BACSU      DHAAYELLKEQLEDVLDTLTDREENVLRLRFGLDDGRTRTLEEVGKVFGVTRERIRQIEA
sp|P00579|RPOD_ECOLI      DSATTESLRAATHDVLAGLTAREAKVLRMRFGIDMNTDYTLEEVGKQFDVTRERIRQIEA
                          * *: * *:    .***  ** ** :***:***:* .   ******* *.***********

                                 600
sp|P06224|SIGA_BACSU      KALRKLRHPSRSKRLKDFLE-
sp|P00579|RPOD_ECOLI      KALRKLRHPSRSEVLRSFLDD
                          ************: *:.**:
```

Numbering with respect to *E. coli* σ^{70}

-35 element contacts

core RNAP recognition

extended -10

-10 element (open complex)

Contacts upstream of the -10 element

-10 element closed complex

Supplementary Figure 5: Alignment of the *B. subtilis* σ^{A} and *E. coli* σ^{70} amino acid sequences.

Supplementary Figure 6

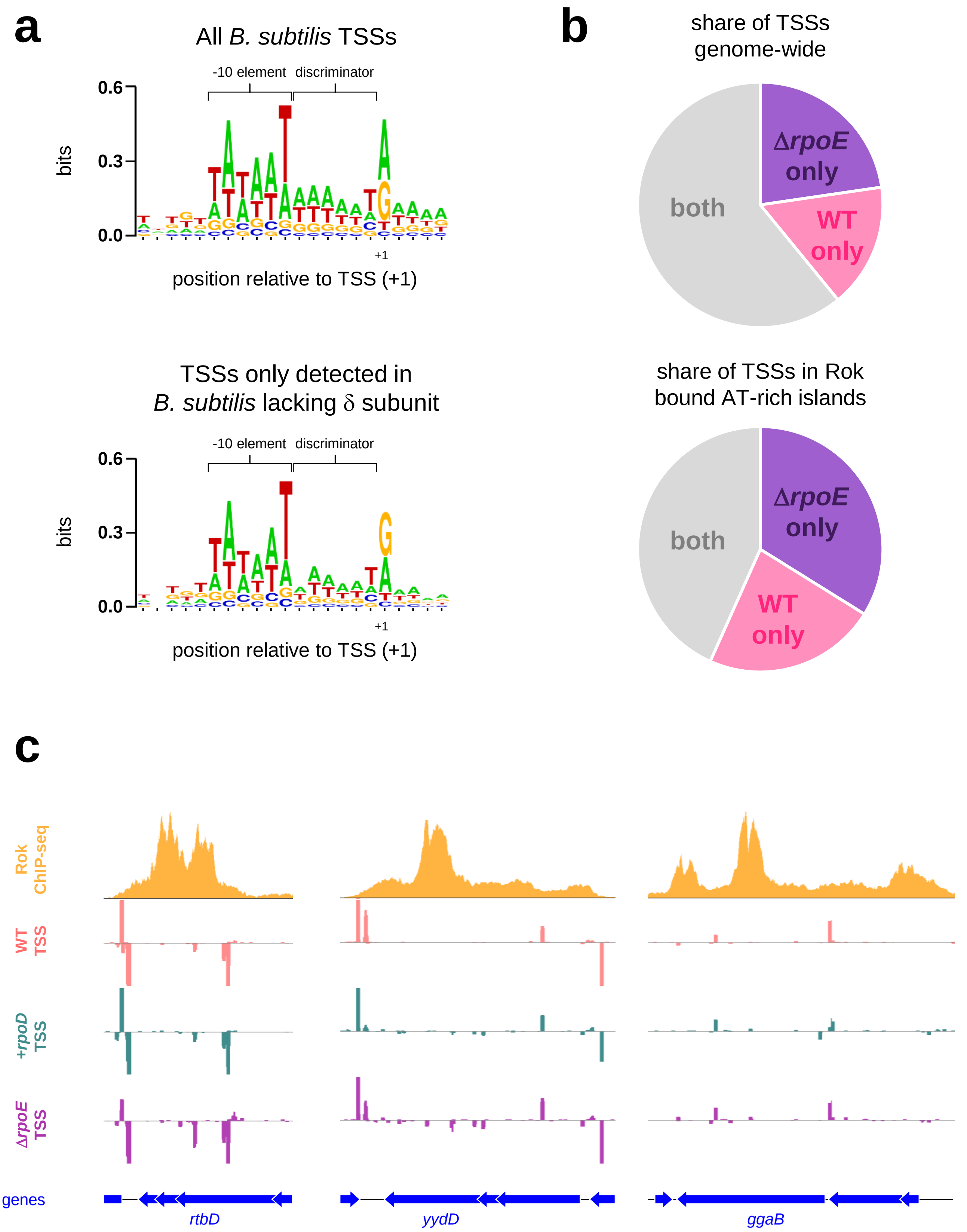

Supplementary Figure 6: Loss of the *B. subtilis* RNA polymerase δ subunit leads to increased promiscuous transcription of horizontally acquired DNA.

a) The panel shows DNA sequence logos generated by aligning nucleic acid regions upstream of all *B. subtilis* transcription start sites (top) and those only detected following deletion of *rpoE* encoding the RNA polymerase δ subunit (bottom). Note the change in sequence at the +1 position.

b) The pie charts show distribution of *B. subtilis* TSSs identified only in the absence of *rpoE*, only in wild type cells, or in both genetic backgrounds. Those TSSs only detected in the absence of *rpoE*, encoding the RNA polymerase δ subunit, are overrepresented in horizontally acquired AT-rich sections of DNA targeted by Rok.

c) Three Rok targeted genomic regions are shown. Data for Rok occupancy are shown by the orange graph[35]. Transcription start sites (TSSs) were identified by cappable-seq for wild type, *rpoD+*, or Δ*rpoE* cells. In the cappable-seq data only RNA 5' ends are sequenced and so the upstream edge of each peak indicates a TSS. Sequence reads mapping to the top and bottom DNA strands are shown above and below the central horizontal line in each plot. Genes are shown by blue arrows.

Supplementary Figure 7

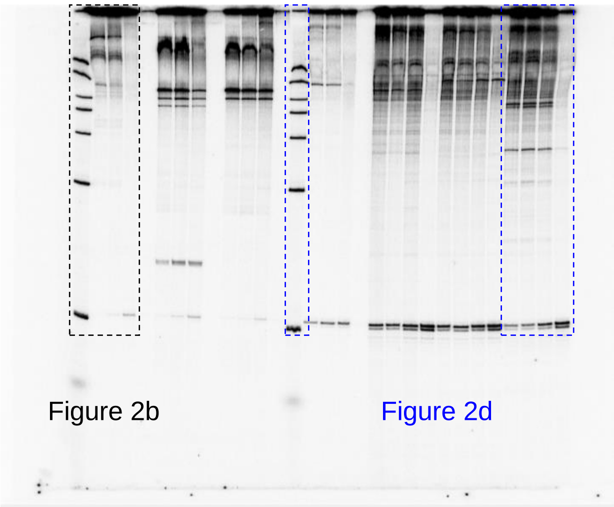

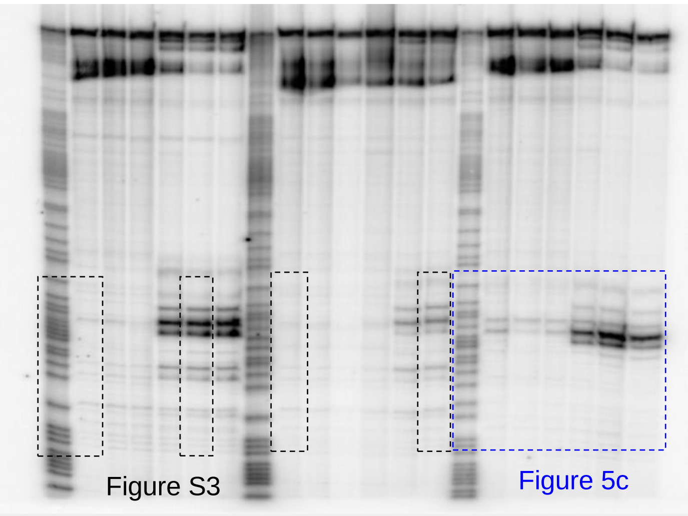

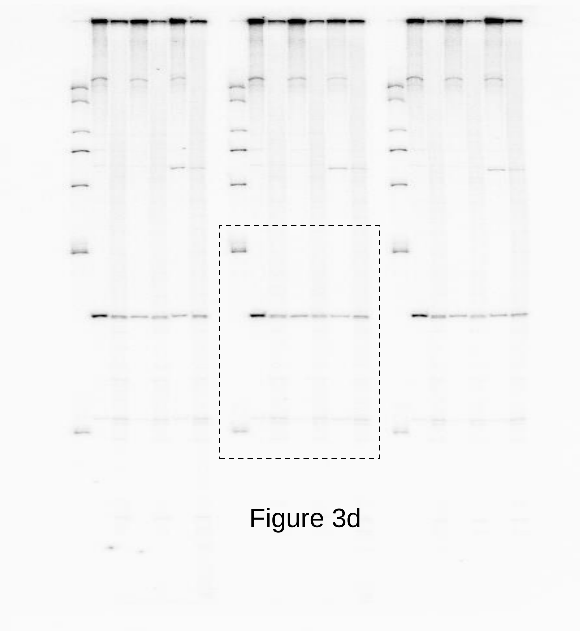

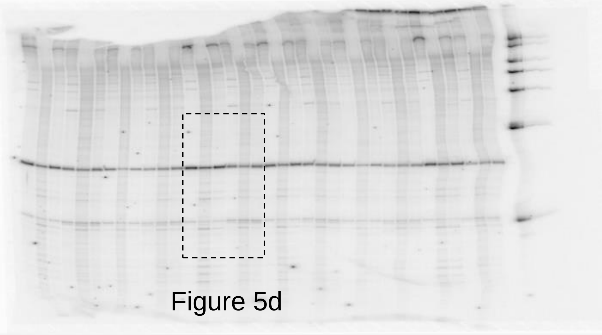

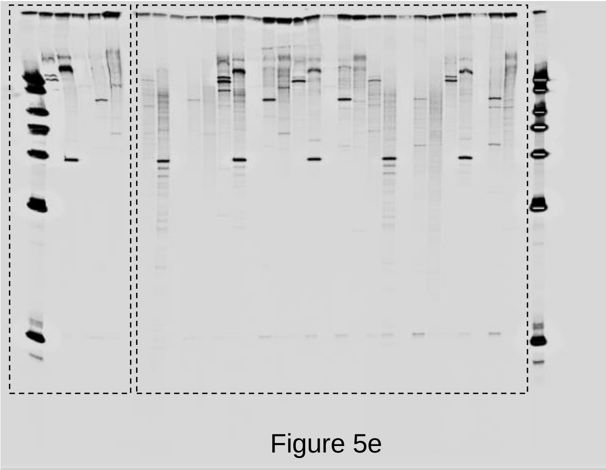

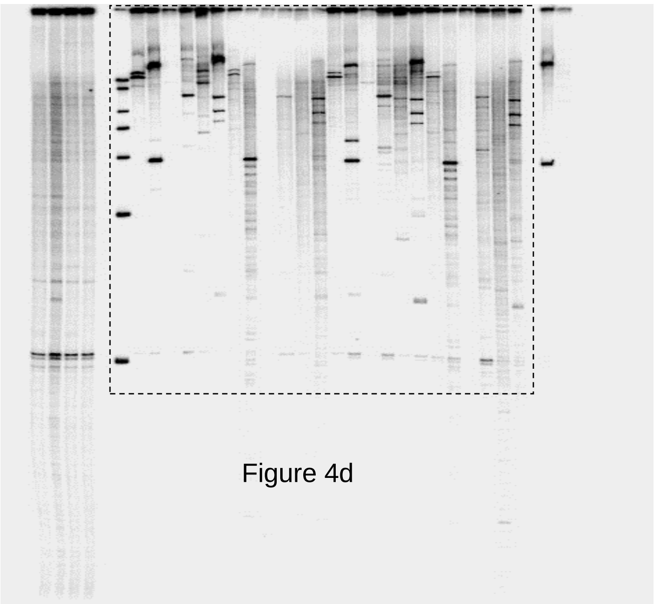

Supplementary Figure 7: Original gel images. Sections of gel images used in figures are boxed, labelled, and where necessary colour coded, for clarity.

Supplementary Table 1: Strains, plasmids, synthesised gene strands and oligonucleotides

**Name**	**Description**	**Source**
	Bacillus subtilis strains	
168ca	*trpC2*	13
Δ*rok*	*trpC2* Δ*rok*::*kan*	14
Δ*rpoE*	*trpC2* Δ*rpoE*::*kan*	14
rpoD+	*trpC2 amyE*::Phyperspank *rpoD* (spec)	This work
	Escherichia coli strains	
DH5α	*fhuA*2 Δ(*argF*-*lacZ*)U169 *phoA glnV*44 Φ80 Δ(*lacZ*)M15 *gyrA*96 *recA*1 *relA*1 *endA*1 *thi*-1 *hsdR*17	NEB
MG1655	K-12 F– λ– *ilvG*– *rfb*-50 *rph*-1	15
Δ*hns*	K-12 F– λ– *ilvG*– *rfb*-50 *rph*-1 Δ*hns*::*kan*	16
	Plasmids	
pSR	pBR322-derived plasmid. Features cloning site upstream λoop transcription terminator. AmpR	17
pDR111	IPTG inducible Phyperspank promoter and specR cassette flanked by amyE sequences for chromosomal integration. AmpR	1
pRok	pUC19 derivative encoding *B. subtilis rok* under the control of the *E. coli hns* promoter. AmpR	This work
pET-28a	For T7 based, IPTG induced expression of N-terminal His tag fusion protein. KanR.	Novagen
pET-21a	For T7 based, IPTG induced expression of C-terminal His tag fusion protein. AmpR.	Novagen
	Synthesised gene fragments (5' to 3')[1]	
pVeg	CACGGCGAATTCTCCATCCTCTCAGAGCTCACATTTATTTGA<br>CAAAAATGGGCTCGTGTTGTACAATAAATGT**A**GTGCTCGCTT<br>TACGTCACCTGCTTCAGCTCTCACATAAGGAGGAACTACCAT<br>GAATGTG*AAGCTT*TTATCC	This study
pVeg AT	CACGGCGAATTCTCCATCCTCTCAGAGCTCACATTTATTTGC<br>TGAAAATGGGCTCGTCTTGTATAATAAAAAT**G**ATGCTCGCTT<br>TACGTCACCTGCTTCAGCTCTCACATAAGGAGGAACTACCAT<br>GAATGTG*AAGCTT*TTATCC	This study
pVeg GC	CACGGCGAATTCTCCATCCTCTCAGAGCTCACATTTATTTGC<br>TGAAAATGGGCTCGTCTTGTATAATGCACGT**G**ATGCTCGCTT	This study

	TACGTCACCTGCTTCAGCTCTCACATAAGGAGGAACTACCATGAATGTG*AAGCTT*TTATCC	
pVeg GC +1bp	CACGGC*GAATTC*TCCATCCTCTCAGAGCTCACATTTATTTGCTGAAAATGGGCTCGTCTTGTATAATCGCACGT**G**ATGCTCGCTTTACGTCACCTGCTTCAGCTCTCACATAAGGAGGAACTACCATGAATGTG*AAGCTT*TTATCC	This study
	Oligonucleotides (5' to 3')[2]	
pSR pVeg F	gaggaactaccatgaatgtgAAGCTTTTATCCACTCCCCATCCCCTCCAGTAATG	This study
pSR pVeg R	gtgagctctgagaggatggaGAATTCGCCGTGTTGAAGACGAAAGGGCCTCGTGA	This study
pSR F_*Hin*dIII	AAGCTTACTCCCCATCCCCTC	This study
pSR R_*Eco*RI	GAATTCTTGAAGACGAAAGGGCC	This study
sunA pSR F	cctttcgtcttcaagaattcTTTTTAAATGGAGCTCAACAATTTATTC	This study
sunA pSR R	aggggatggggagtaagcttTTATCTGCAGAATTGACG	This study
agaB pSR F	atcacgaggccctttcgtcttcaagaattcCACAAAAGTGAACGTTGCCAC	This study
agaB pSR R	tcattactggaggggatggggagtaagcttTTAGTCAGGGATTTGTTCTTTTTG	This study
gmuB pSR F	gaggccctttcgtcttcaagaattcAGGAAAACTTTGAAATTCATAAC	This study
gmuB pSR R	actggaggggatggggagtaagcttTTATTGATTCACCATTAAGGAC	This study
pSR F_*Hin*dIII	ggaacggtattagaagcttACTCCCCATCCCCTC	This study
pSR R_*Xho*I	caaaaaacaactcgagTTGAAGACGAAAGGGCCT	This study
comK pSR F	cgtcttcaactcgagTTGTTTTTTGCGTGTTGCGG	This study
comK pSR R	gggagtaagcttCTAATACCGTTCCCCGAGCTC	This study
pSR F_*Xho*I	CTCGAGACTCCCCATCCCCTCCAGT	This study
pSR R_*Eco*RI	GAATTCTTGAAGACGAAAGGGCC	This study
qoxA pSR F	cctttcgtcttcaagaattcAAAATGAAATTTTTGATTGACCTAAG	This study
qoxA pSR R	aggggatggggagtctcgagTCATTCTTCTGTATCATCAGACTTC	This study
yddK pSR F	cctttcgtcttcaagaattcTACATTAATTGTATTATGTCAGAATAAC	This study

yddK pSR R	aggggatggggagtctcgagTTATTTTAATTTCGTATTAAATTTAGCC	This study
ynaB pSR F	cctttcgtcttcaagaattcAGGATTGGGTTTCCTTATGGCTCAAC	This study
ynaB pSR R	aggggatggggagtctcgagTATTCTGAAACCAGTTTATAATACTCTTGGAGATTC	This study
yydD pSR F	cctttcgtcttcaagaattcGTGATTGTTATGATTATAAAAAATCTTTTTG	This study
yydD pSR R	aggggatggggagtctcgagTCAAAATCTAAAACCAAAAAATCTATTTTTATC	This study
pET21/*rok* F	atcagcaaacgaactcgagCACCACCACCACCACCACTGAGATC	This study
pET21/*rok* R	tttcattaaacatggatccGCGACCCATTTGCTGTCCACCAGT	This study
rok F	ggtcgcggatccATGTTTAATGAAAGAGAAGCTTTGCGCTTG	This study
rok R	gtggtgctcgagTTCGTTTGCTGATTCTGCAGATTCGATTTC	This study
pDR111 F	ctggacgattaaAGTCGACAGCTAGCCGCATG	This study
pDR111 R	gttttgctccatagtagttcctccttatgtAAGCTTAATTGTTATCCGCTCACAATTACAC	This study
rpoD F	aacaattaagcttacataaggaggaactactATGGAGCAAAACCCGCAGTC	This study
rpoD R	ctagctgtcgactTTAATCGTCCAGGAAGCTACG	This study
H-NS promoter F	GAGCTCGGTACCCGGGGATCttctggctaattttatgaaa	This study
H-NS promoter R	GCTTCTCTTTCATTAAACATtgtagtaatctcaaacttat	This study
rok.1 F	ataagtttgagattactacaATGTTTAATGAAAGAGAAGC	This study
rok.1 R	caagtgcaatctacaaaagaTTATTCGTTTGCTGATTCTG	This study
pUC19 F	tttcataaaattagccagaaGATCCCCGGGTACCGAGCTC	This study
pUC19 R	CAGAATCAGCAAACGAATAAtcttttgtagattgcacttg	This study

[1]Restriction sites are italicised. Promoter -35 and -10 elements are underlined.

[2]Optimal ribosome binding sites introduced by the oligonucleotides are underlined. Lowercase sequence denotes complementary sequences for Gibson assembly.
