# Supplementary material for: Xenogeneic silencing strategies in bacteria are dictated by RNA polymerase promiscuity
Source: Nat Commun. 2022 Mar 3;13:1149. doi: 10.1038/s41467-022-28747-1 (PMC8894471; doi:10.1038/s41467-022-28747-1)
Supplement: Supplementary file 2 — Reporting Summary [file 41467_2022_28747_MOESM2_ESM.pdf]

nature portfolio

Corresponding author(s): David Grainger

Last updated by author(s): Feb 4th 2022

Reporting Summary

Nature Portfolio wishes to improve the reproducibility of the work that we publish. This form provides structure for consistency and transparency in reporting. For further information on Nature Portfolio policies, see our Editorial Policies and the Editorial Policy Checklist.

Statistics

For all statistical analyses, confirm that the following items are present in the figure legend, table legend, main text, or Methods section.

n/a	Confirmed	
☐	☒	The exact sample size (n) for each experimental group/condition, given as a discrete number and unit of measurement
☒	☐	A statement on whether measurements were taken from distinct samples or whether the same sample was measured repeatedly
☐	☒	The statistical test(s) used AND whether they are one- or two-sided<br>*Only common tests should be described solely by name; describe more complex techniques in the Methods section.*
☒	☐	A description of all covariates tested
☒	☐	A description of any assumptions or corrections, such as tests of normality and adjustment for multiple comparisons
☐	☒	A full description of the statistical parameters including central tendency (e.g. means) or other basic estimates (e.g. regression coefficient) AND variation (e.g. standard deviation) or associated estimates of uncertainty (e.g. confidence intervals)
☐	☒	For null hypothesis testing, the test statistic (e.g. F, t, r) with confidence intervals, effect sizes, degrees of freedom and P value noted<br>*Give P values as exact values whenever suitable.*
☒	☐	For Bayesian analysis, information on the choice of priors and Markov chain Monte Carlo settings
☒	☐	For hierarchical and complex designs, identification of the appropriate level for tests and full reporting of outcomes
☒	☐	Estimates of effect sizes (e.g. Cohen's d, Pearson's r), indicating how they were calculated

Our web collection on statistics for biologists contains articles on many of the points above.

Software and code

Policy information about availability of computer code

Data collection	Gel images were collected with Quantity One (Biorad) software (version 4.6.9).
Data analysis	Gel images were analysed with Quantity One (Biorad) software. Sequence reads (FASTQ format) were mapped using Bowtie2 (Galaxy version 2.4.2). Coverage for each genome position was extracted from resulting Binary Alignment Map (BAM) files using the genomcov function of BedTools (Galaxy version 2.30.0). The R package GenomicRanges (version 1.44) was used to identify TSSs in different genomic contexts. FeatureCounts (version 2.6) of the Rsubread package (version 2.6) was used to determine gene read counts, which were inputted into the exact function of edgeR (Galaxy version 3.34.0) to determine differential gene expression. DNA sequence logos were generated with WebLogo (version 2.8.2). Other data analysis tasks were completed using Microsoft Excel 2016.

For manuscripts utilizing custom algorithms or software that are central to the research but not yet described in published literature, software must be made available to editors and reviewers. We strongly encourage code deposition in a community repository (e.g. GitHub). See the Nature Portfolio guidelines for submitting code & software for further information.

Data

Policy information about availability of data

All manuscripts must include a data availability statement. This statement should provide the following information, where applicable:

- Accession codes, unique identifiers, or web links for publicly available datasets
- A description of any restrictions on data availability
- For clinical datasets or third party data, please ensure that the statement adheres to our policy

The raw RNA-seq and cappable-seq data generated in this study have been deposited in ArrayExpress under accession code E-MTAB-10777 (https://www.ebi.ac.uk/arrayexpress/experiments/E-MTAB-10777/). Reference genomes NC000964.3 (https://www.ncbi.nlm.nih.gov/nuccore/NC_000964.3) or U00096.3 (https://

www.ncbi.nlm.nih.gov/nuccore/545778205) were used as appropriate. Results generated from processing of the sequencing data (e.g. to determine changes in gene expression or TSS signals) are available as Source Data or Supplementary Data files. The ChIP-seq data used were obtained from the ArrayExpress or NCBI databases for E. coli H-NS (E-MTAB-332, https://www.ebi.ac.uk/arrayexpress/experiments/E-MTAB-332/) or B. subtilis Rok (PRJNA272948, https://www.ncbi.nlm.nih.gov/bioproject/?term=PRJNA272948) respectively. All Original gel images are in Supplementary Figure 7 and results obtained from quantification of band intensities are provided as Source Data.

Field-specific reporting

Please select the one below that is the best fit for your research. If you are not sure, read the appropriate sections before making your selection.

☒ Life sciences ☐ Behavioural & social sciences ☐ Ecological, evolutionary & environmental sciences

For a reference copy of the document with all sections, see nature.com/documents/nr-reporting-summary-flat.pdf

Life sciences study design

All studies must disclose on these points even when the disclosure is negative.

Sample size	Sample sizes were chosen to permit appropriate downstream statistical testing and in line with normal expectations for the field. For instance, cappable-seq assays were done twice, as described by the authors that first described the approach (Ettwiller, L., Buswell, J., Yigit, E. & Schildkraut, I. A novel enrichment strategy reveals unprecedented number of novel transcription start sites at single base resolution in a model prokaryote and the gut microbiome. BMC Genomics 17, 199 (2016)).
Data exclusions	No data were excluded.
Replication	The cappable-seq and RNA-seq experiments were done twice to confirm reproducibility. Biochemical experiments (Potassium permanganate footprints and in vitro transcription assays) were done at least twice to confirm reproducibility. For such experiments, three replicates were done if subsequent statistical analysis was applied. In many cases we used independent methods to confirm our findings. All attempts at replication were successful.
Randomization	Not applicable since samples were not allocated to experimental groups.
Blinding	Not applicable since samples were not allocated to experimental groups.

Reporting for specific materials, systems and methods

We require information from authors about some types of materials, experimental systems and methods used in many studies. Here, indicate whether each material, system or method listed is relevant to your study. If you are not sure if a list item applies to your research, read the appropriate section before selecting a response.

Materials & experimental systems

n/a	Involved in the study	
☒	☐	Antibodies
☒	☐	Eukaryotic cell lines
☒	☐	Palaeontology and archaeology
☒	☐	Animals and other organisms
☒	☐	Human research participants
☒	☐	Clinical data
☒	☐	Dual use research of concern

Methods

n/a	Involved in the study	
☒	☐	ChIP-seq
☒	☐	Flow cytometry
☒	☐	MRI-based neuroimaging
