# Supplementary material for: Xenogeneic silencing strategies in bacteria are dictated by RNA polymerase promiscuity
Source: Nat Commun. 2022 Mar 3;13:1149. doi: 10.1038/s41467-022-28747-1 (PMC8894471; doi:10.1038/s41467-022-28747-1)
Supplement: Supplementary file 4 — Description of Additional Supplementary Files [file 41467_2022_28747_MOESM4_ESM.pdf]

Title: Supplementary Data 1.
Description: Positions of transcription start sites (TSSs) in E. coli and B. subtilis strains. Numbers refer to the chromosomal position and + or - symbols the DNA strand. Strains are indicated.

Title: Supplementary Data 2.
Description: Changes in gene expression detected by RNAseq in Escherichia coli or Bacillus subtilis lacking hns or rok respectively.

Title: Supplementary Data 3.
Description: Boundaries of genomic regions classed as HNS bound, Rok, bound, AT-rich Islands, or horizontally acquired.
